# Preserving Derivative Information while Transforming Neuronal Curves

**Published:** 2023-03-16

**Authors:** Thomas L. Athey, Daniel J. Tward, Ulrich Mueller, Laurent Younes, Joshua T. Vogelstein, Michael I. Miller

**Affiliations:** 1 Department of Biomedical Engineering, Johns Hopkins University, Baltimore, MD, USA; 2 Institute of Computational Medicine, Johns Hopkins University, Baltimore, MD, USA; 3 Department of Computational Medicine, University of California at Los Angeles, Los Angeles, CA, USA; 4 Department of Neurology, University of California at Los Angeles, Los Angeles, CA, USA; 5 Department of Neuroscience, Johns Hopkins University, Baltimore, MD, USA; 6 Department of Applied Mathematics & Statistics, Johns Hopkins University, Baltimore, MD, USA; 7 Center for Imaging Science, Johns Hopkins University, Baltimore, MD, USA; 8 Kavli Neuroscience Discovery Institute, Johns Hopkins University, Baltimore, MD, USA

## Abstract

The international neuroscience community is building the first comprehensive atlases of brain cell types to understand how the brain functions from a higher resolution, and more integrated perspective than ever before. In order to build these atlases, subsets of neurons (e.g. serotonergic neurons, prefrontal cortical neurons etc.) are traced in individual brain samples by placing points along dendrites and axons. Then, the traces are mapped to common coordinate systems by transforming the positions of their points, which neglects how the transformation bends the line segments in between. In this work, we apply the theory of jets to describe how to preserve derivatives of neuron traces up to any order. We provide a framework to compute possible error introduced by standard mapping methods, which involves the Jacobian of the mapping transformation. We show how our first order method improves mapping accuracy in both simulated and real neuron traces, though zeroth order mapping is generally adequate in our real data setting. Our method is freely available in our open-source Python package brainlit.

## Main

The brain functions as a network of chemical and electrical activity, so identifying how neurons connect across brain regions is central to understanding how the brain works, and how to treat brain diseases. Modern neuroscience techniques can image single neuron morphology at scale [7], and subsequent neuron tracing can help discover new morphological subtypes [21]. Due to anatomical variation, and deformations that may have occurred during tissue preparation, neuron traces need to be mapped between coordinate spaces to compare morphologies from different brain samples. Brain registration software often includes neuron mapping implementations, but these implementations have not been thoroughly characterized from a numerical analysis perspective.

This question is relevant to the ongoing work of the international neuroscience community, including the Brain Initiative Cell Census Network (BICCN), to establish comprehensive neuronal atlases of the mammalian brain [3]. This effort has produced many images of stained or fluorescently labeled brains, which are being used to generate digital neuron traces for morphological analysis. The traces are commonly stored as a set of connected 3D coordinates, or knots, such as in the SWC format [5, 17]. The connections between the knots are classically represented as cylinders [5], or conical frustums [13], but here we ignore radius information, since it is not generated by all neuron tracing methods. Consequently, the whole neuron trace is considered to be a tree of piecewise linear curves.

**Figure 1:**
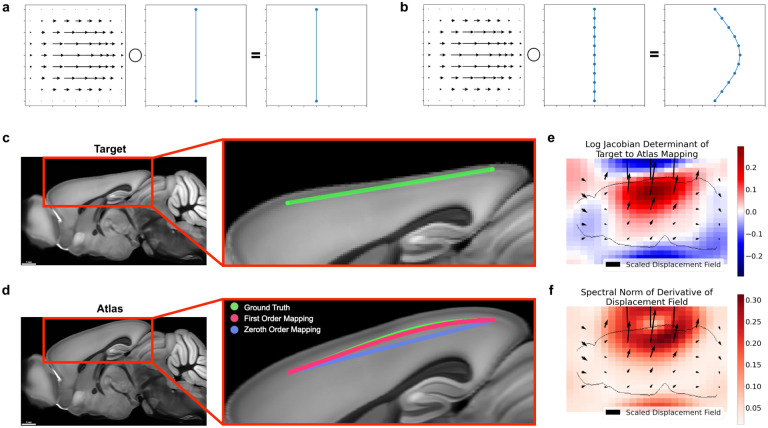
Neglecting the action of a nonlinear mapping on a curve’s derivatives can introduce errors. **a-b** Different samplings of a curve can lead to different results under nonlinear deformations, such as only sampling the endpoints (**a**) versus sampling several times along the curve (**b**). **c-d** Large distances between control points can contribute to mapping inaccuracies. The green line segment following cortical layers 2/3 in a synthetic mouse brain image (**c**) is defined only by its endpoints. Transforming only the positions of the endpoints (zeroth order mapping, **d**), is less accurate than incorporating the action on the derivatives as well (first order mapping, **d**). **e-f** Quantitative descriptions of the mapping from target to atlas via the logarithm of the Jacobian determinant, which quantifies expansion and compression (**e**), and the spectral norm of the displacement field, which plays a role in an error bound of zeroth order mapping (**f**).

In order to assemble these traces into a complete picture of the various neuron morphologies in the brain, scientists need a way to map neuron traces into common coordinate systems. Several popular software applications exist for this task and are used to assemble atlases of neuron morphology. For example, Peng et al. [15] used mBrainAligner [16], Gao et al. [9] used the Computational Morphometry Toolkit, and the MouseLight project [21] used displacement fields from Fedorov et al. [8]. Existing methods use what we call *zeroth order* curve mapping in that they only map the positions of the knots. However, depending on the nonlinearity of the mapping, and the continuous representation of the neuron trace, zeroth order mapping is sensitive to different samplings of the original neuronal curve (Fig. 1a,b). In other words, sampling the same curve different ways while tracing in the original image may lead to different mapped morphologies. It is critical that neuron mapping methods preserve the geometry of digital neuron traces in order to build reliable atlases of neuron morphology, and to accurately identify deviations in diseased brains.

In this work, we introduce a method to preserve derivative information when mapping neuronal curves, and investigate the conditions under which this technique is advantageous to existing methods (Figure 1). We applied our method to both simulated data and real neuron traces from a whole mouse brain image, and the code used developed in this work is freely available in our Python package brainlit.

## Results

### Action of Diffeomorphisms on Discrete Samplings

In the following sections, we use *C*^*k*^ to represent the space of continuous functions with *k* continuous derivatives, where the domain and range can be inferred by the context. We model a neuronal process as a regular 3D curve $c:[0,L]\to {\mathbb{R}}^{3}$, *c* ∈ *C*^*k*^, and $|\dot{c}|>0$. When neurons are traced, they are typically stored as a sequence of points ${\left\{x_{i}=c\left(t_{i}\right):t_{i}<t_{i+1}\right\}}_{i=1}^{n}$, where the independent variables *t*_*i*_ are not specified and, from a geometric point of view are arbitrary [22]. When there is a diffeomorphism between coordinate systems $\phi :{\mathbb{R}}^{3}\to {\mathbb{R}}^{3}$, these traces are mapped via the group action:

$$\phi \cdot {\left\{x_{i}\right\}}_{i=1}^{n}={\left\{\phi \left(x_{i}\right)\right\}}_{i=1}^{n}$$

We want to extend the space of traces, and the associated action, to include derivatives of the underlying curve denoted *∂*_*t*_*c*. This can be done using the jet space *J*^*k*^. In our setting, *J*^*k*^ = [0, *L*] × *X*^(*k*)^, where an element of *X*^(*k*)^ is a *k* + 1-tuple $\left(x^{0},x^{1},\ldots ,x^{k}\right)\in {\left({\mathbb{R}}^{3}\right)}^{k+1}$ representing a position and first *k* derivatives of a curve in ${\mathbb{R}}^{3}$. A *C*^*k*^ curve $c:[0,L]\to {\mathbb{R}}^{3}$ can be extended to a curve $\hat{c}:[0,L]\to X^{\left(k\right)}$ simply by adding derivatives, with $\hat{c}(t)=\left(c(t),\partial_{t}c(t),\ldots ,\partial_{t}^{k}c(t)\right)\in X^{\left(k\right)}$ [12].

The *C*^*k*^ diffeomorphisms have a natural group action on the jet space *J*^*k*^, ensuring the commutation between the standard action of diffeomorphisms on curves, (*ϕ*, *c*) ↦ *ϕ* ο *c* and their extensions, such that the identity $\phi \cdot \hat{c}(t)=\hat{\phi οc}(t)$ holds for all curves *c* and times *t*, defining the left-hand side. For example, for *k* = 2, this provides

$$\phi \cdot \left(t,x^{0},x^{1},x^{2}\right)=\left(t,\phi \left(x^{0}\right),D\phi \left(x^{0}\right)x^{1},D\phi \left(x^{0}\right)x^{2}+D^{2}\phi \left(x^{0}\right)\left(x^{1},x^{1}\right)\right)$$

Neuron traces, as mentioned before, involve a sequence of samples with time-stamps ${\left\{\left(t_{i},x_{i}^{\left(k\right)}\right)\right\}}_{i=1}^{n}$, identified as elements of (*J*^*k*^)^*n*^, the *n*-fold Cartesian product of *J*^*k*^. Our diffeomorphisms will act on such a sequence as follows:

**Statement 1.** *For a sequence of time-stamped elements on the jet space*, $T={\left\{\left(t_{i},x_{i}^{\left(k\right)}\right)\right\}}_{i=1}^{n}\text{ in }{\left(J^{k}\right)}^{n}$, *we define the action of diffeomorphisms*

(1) $$\phi \cdot T={\left\{\left(t_{i},\phi \cdot x_{i}^{\left(k\right)}\right)\right\}}_{i=1}^{n}$$

The fact that this operation provides an action is is an established result [12], and the proof is provided in the Supplement. We will define *k’th order discrete mapping* to be the action in Equation 1 of a diffeomorphism on a curve sampling that includes *k* derivatives. The axioms that define group actions are important to verify because they ensure that applying the identity transformation does not change the object, and that applying a composition of transformations is equivalent to applying the individual transformations successively [4]. Further, group actions can exchange mathematical structure between the acting group and the set being acted upon, and they are at the core of several important theorems [18].

The *k*’th order discrete mapping method allows us to compute the first *k* derivatives of the transformed curve. We will interpolate the transformed curve using splines of order 2*k*+1 that satisfy the derivative values. For example, zeroth order mapping will produce a first order spline and first order mapping will produce a cubic Hermite spline [10].

### Error Analysis of Zeroth and First Order Mapping

Now we will examine the error introduced by zeroth order mapping, which is used by existing neuron mapping methods. First, note that under affine transformations, zeroth order mapping of piecewise linear curves introduce no error, so these results are most applicable under non-affine transformations. The following results require that the curve *c* be parameterized by arc length. However, all continuously differentiable regular curves can be reparameterized by arc length [22]. We use |·| to denote the Euclidean norm for elements of ${\mathbb{R}}^{d}$, and the spectral norm for matrices.

**Proposition 1. [*Zeroth Order Mapping Error Bound 1*]** *Say* $\phi :{\mathbb{R}}^{3}\to {\mathbb{R}}^{3}$ *is a C*^1^ *diffeomorphism and* $c\text{ : }[0,L]\to {\mathbb{R}}^{3}$ *is a continuous*, *piecewise C*^1^ *curve parameterized by arc length with knots* $\{t_{i}:t_{1}=0,t_{n}={L,t_{i-1}<t_{i}\}}_{i=1}^{n}$. *For the transformed curve f* = *ϕ* ο *c*, *the zeroth order mapping defines a first order spline g which satisfies:*

(2) $$\underset{{}_{t\in [0,L]}}{\text{max}}|f(t)-g(t)|\leq \delta \sqrt{3}\underset{{}_{t\in [0,L]}}{\text{max}}|D\phi οc(t)|$$

*where δ* ≜ max_2≤*i*≤*n*_ |*t*_*i*_ − *t*_i−1_|, *and Dϕ* ο *c*(*t*) *is the Jacobian of ϕ evaluated at c*(*t*).

The proof of the proposition is an adaptation of a spline approximation theorem in [10]. In practice, the neuronal curves that are being mapped (*c* → *ϕ* ο *c*) are often piecewise linear curves themselves. So, we present another bound in this setting, and when *ϕ* is close to the identity transformation.

**Proposition 2. [*Zeroth Order Mapping Error Bound 2*]** *Say* $\phi :{\mathbb{R}}^{3}\to {\mathbb{R}}^{3}$ *is a C*^1^ *diffeomorphism and* $c:[0,L]\to {\mathbb{R}}^{3}$ *is a continuous*, *piecewise linear curve parameterized by arc length with knots* $\{t_{i}:t_{1}=0,t_{n}={L,t_{i-1}<t_{i}\}}_{i=1}^{n}$. *For the transformed curve f* = *ϕ* ο *c*, *the zeroth order mapping defines a first order spline g which satisfies:*

(3) $$\underset{t\in [0,L]}{\text{max}}|f(t)-g(t)|\leq \underset{{}_{i\in \{0,\ldots ,n\},t\in \left[t_{i-1},t_{i}\right]}}{\text{max}}\frac{1}{2}\left(\left|D\phi οc(t)-I||t_{i}-t_{i-1}\right|+\left|\epsilon_{i}-\epsilon_{i-1}\right|\right)$$

*where* $\epsilon_{i}≜c\left(t_{i}\right)-\phi \left(c\left(t_{i}\right)\right)$ *and Dϕ* ο *c*(*t*) *is the Jacobian of ϕ evaluated at c*(*t*).

In summary, we have presented two bounds on the maximum deviation of the true transformed curve from the approximation obtained by zeroth order mapping. Eq. 2 applies to all piecewise differentiable curves but involves |*Dϕ*| which does not vanish even if *ϕ* is the identity map (in which case zeroth order mapping has zero error for piecewise linear curves). Eq. 3 only applies to piecewise linear *c*, but goes to zero as *ϕ* approaches the identity map. Both bounds depend on the spectral norm of *Dϕ*, and arc lengths of the original curve segments. We note that log|*Dϕ*| is the finite time Lyapunov exponent, a well-known quantity in field dynamics which characterizes the amount of stretching in a differentiable flow.

It is worth noting that Eq. 2 and 3 apply to max_*t*∈[0,*L*]_ |*f*(*t*) − *g*(*t*)|, which is not parameterization invariant, and therefore not a strictly geometric quantity. However we note that this quantity is an upper bound of the Frechet distance, which is parameterization invariant.

In this paper we demonstrate first order mapping in an effort to mitigate this mapping error. Such a method has the advantage of having superior error convergence at the knots as a consequence of Taylor’s theorem. Further, we present a set of error bounds that helps clarify the advantage of first order mapping.

**Proposition 3. [*Comparable Bounds for Zeroth and First Order Mapping*]** *Say $\phi :{\mathbb{R}}^{3}\to {\mathbb{R}}^{3}$ is a C*^4^ *diffeomorphism and $c:[a,b]\to {\mathbb{R}}^{3}$ is a continuous*, *piecewise C*^4^ *curve parameterized with knots* $\{t_{i}:t_{1}=a,t_{n}={b,t_{i-1}<t_{i}\}}_{i=1}^{n}$. *For the transformed curve f* = *ϕ* ο *c defined by coordinate functions f* = (*f*^0^, *f*^1^, *f*^2^)^*T*^, *the zeroth order mapping defines a first order spline g*_0_ *which satisfies:*

(4) $$\underset{t\in [a,b]}{\text{max}}\left|f(t)-g_{0}(t)\right|\leq \frac{\sqrt{3}}{4}\underset{{}_{t\in [a,b],j\in \{0,1,2\}}}{\text{max}}\left|\partial_{t}^{\left(4\right)}f^{j}(t)\right|{\left(\frac{\delta }{2}\right)}^{4}+\;\frac{\sqrt{3}}{2}{\left(\frac{\delta }{2}\right)}^{2}\underset{i\in \{1\ldots n\},j\in \{0,1,2\}}{\text{max}}\left|\partial_{t}^{\left(3\right)}f^{j}\left(t_{i}\right)\right|\left(\frac{\delta }{2}\right)+\;\frac{\sqrt{3}}{2}{\left(\frac{\delta }{2}\right)}^{2}\underset{i\in \{1\ldots n\},j\in \{0,1,2\}}{\text{max}}\left|\partial_{t}^{\left(2\right)}f^{j}\left(t_{i}\right)\right|$$

*where δ* ≜ max_2≤*i*≤*n*_ |*t*_*i*_ − *t*_*i*−1_| *and* $\partial_{t}^{\left(k\right)}f^{j}(t)$ *is the k’th derivative of f*^*j*^ *evaluated at t*. *Also*, *the first order mapping defines a third order spline g*_1_, *which satisfies*

(5) $$\underset{t\in [a,b]}{\text{max}}\left|f(t)-g_{1}(t)\right|\leq \frac{\sqrt{3}}{4!}\underset{t\in [a,b],j\in \{0,1,2\}}{\text{max}}\left|\partial_{t}^{\left(4\right)}f^{j}(t)\right|{\left(\frac{\delta }{2}\right)}^{4}$$

*and we note that the bound in 5 is tighter than the bound in 4*. *Further*, *there exists a transformed curve f and a set of knots* ${\left\{t_{i}\right\}}_{i=1}^{n}$ *that achieves both bounds exactly*.

Thus, we present related error bounds on both zeroth and first order mapping methods, both of which are achieved for some curve. The error bound for first order mapping is smaller than that for zeroth order mapping, though for any given curve, either method may produce smaller error than the other. Proofs for the propositions are in the supplement.

### Software Implementation

We implemented a first order discrete mapping method in our our open-source Python package brainlit. In accordance with original SWC formulation [5, 17], we compute one-sided derivatives at the knots of the curve from first order splines. Then, once the knot positions and derivatives are transformed, we generate a continuous curve in the new space using Hermite interpolation. Further details of our implementation can be found in the Methods.

**Figure 2:**
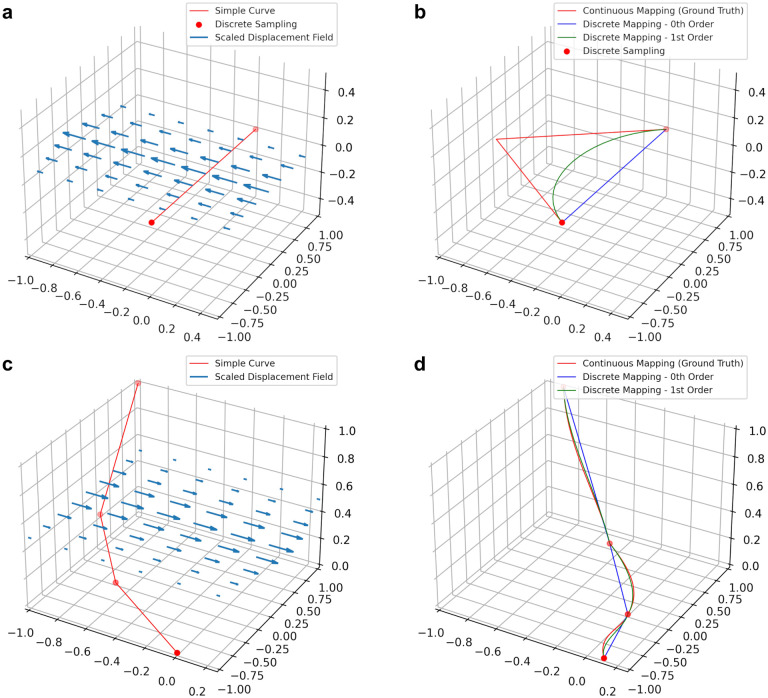
Preserving derivative information can mitigate errors when transforming discretized curves. **a-b** Applying a nonlinear deformation field to a single line segment (**a**) using zeroth and first order mapping (**b**). **c-d** Applying a nonlinear deformation field to a piecewise linear curve (**c**) using zeroth and first order mapping (**d**). Zeroth and first order discrete mapping methods are shown relative to ground truth considered to be the application of the vector field to a dense sampling of the original curves.

Figure 2 shows examples of our method on simulated data, compared to the zeroth order method, and the “ground truth” where we map a dense sampling of points along the first order spline of the original curve.

### Application to Real Neurons

**Figure 3:**
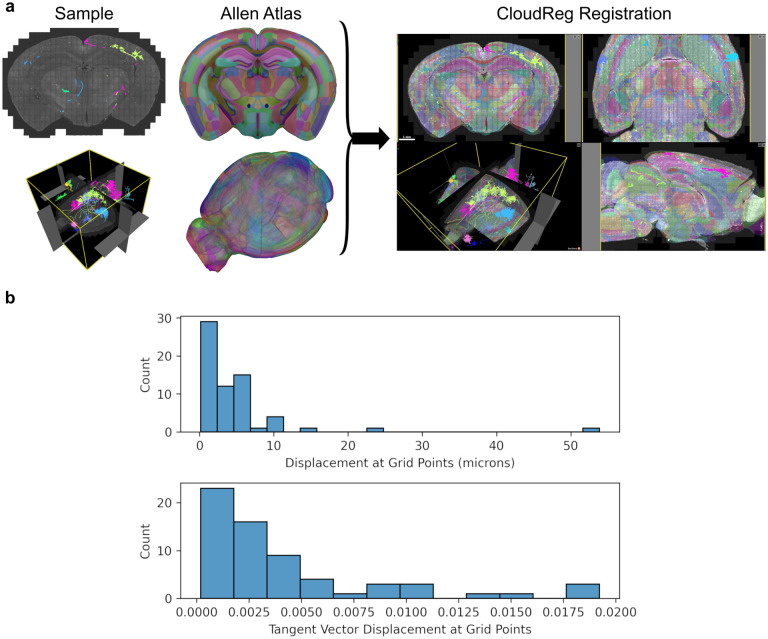
Registration of whole-brain image from Janelia MouseLight project to Allen Reference Atlas (ARA) [20]. **a**, The whole brain image (shown under “Sample” with reconstructed neurons in color) was registered to the ARA using CloudReg [6] (**a**). **b** Histograms of position displacement magnitudes and displacement magnitude of the tangent vector (1, 1, 1)^*T*^ by the LDDMM component of the registration across a regular 3D grid over the sample with 100 micron spacing.

**Figure 4:**
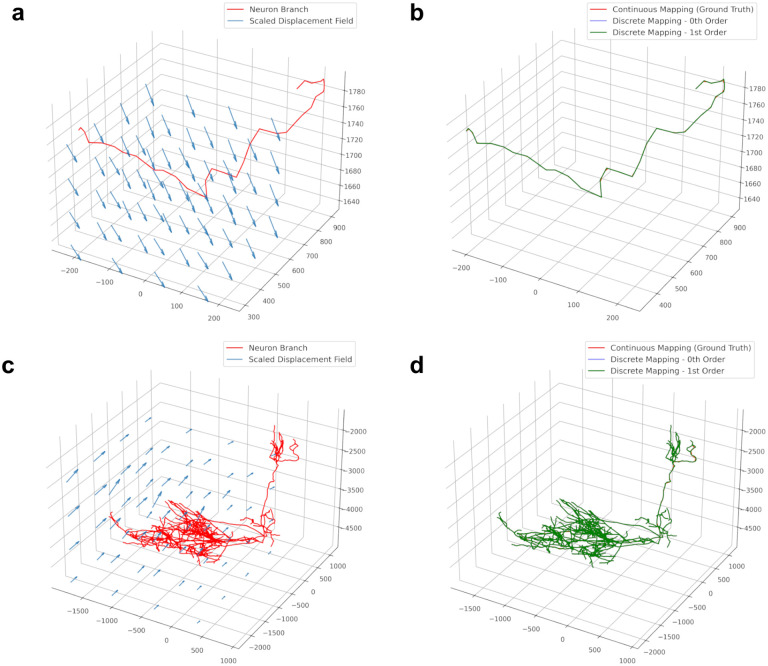
Deformation of reconstructed mouse neurons by a transformation derived from image based registration. **a-b** Single branch of a neuron reconstruction before (**a**) and after (**b**) applying the LDDMM component of the registration. **a-b** Full neuron reconstruction before (**a**) and after (**b**) applying the LDDMM component of the registration. Axes units are in microns.

We applied our method to reconstructed neurons in SWC format from a whole mouse brain image from the Janelia MouseLight project [21]. Neurons have a tree-like structure, and we split them into non-branching curves in order to apply our mapping methods. We follow a method introduced previously [1] where the root to leaf path with the longest arc length is recursively removed until the tree is reduced to non-bifurcating “branches”. The whole-brain image was registered to the Allen Reference atlas [20] using CloudReg [6] (Figure 3). CloudReg computes a transformation that is decomposed into an affine component, and nonlinear large deformation diffeomorphic metric mapping (LDDMM) component. Zeroth and first order mapping under the LDDMM component was applied to the reconstructed neurons (Figure 4). The registration transformation for this sample had only a small LDDMM component, as evidenced by the small displacement values in Figure 3b. As in Figure 2, we consider ground truth to be the “continuous mapping” of the original piecewise linear curve where each line segment is densely sampled, and all the points are mapped into that atlas space.

**Figure 5:**
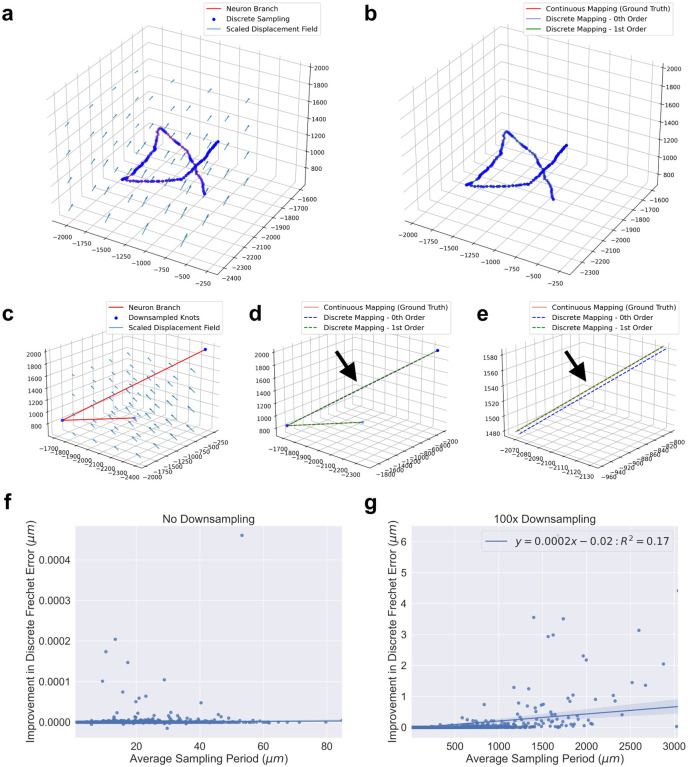
Application of discrete mapping methods to original and downsampled neuron traces. **a-b**, Applying the brain registration vector field to a neuron branch trace **(a)** where both zeroth order and first order mapping remain close to ground truth **(b)**. **c-e**, Applying the same vector field to the trace after downsampling **(c)** where the first order mapping is more accurate along a long segment of the branch (**d**, black arrow) which is more visible in a closer view (**e**, black arrow). **f-g**, Comparison of discrete Frechet error between zeroth and first order mapping methods from ground truth for 22,984 neuron branches (positive values indicate zeroth order mapping had more error). On the original neuron traces, zeroth order and first order mapping had very similar error **(f)**. When the traces were downsampled by 100 times, first order mapping often performed better with a weak positive correlation between difference of error and average sampling period **(g)**. Axes units in **a-e** are microns.

In our experiment, we observed that both zeroth and first order mapping agreed with our ground truth. The overlap of the curves in Figure 5b is an example of both methods being accurate. However, when we downsampled the neuron branch traces 100 times, then first order mapping was more similar to ground truth, as in Figure 5d–e. Both zeroth and first order mapping methods were given the same amount of information, the positions of the knots.

We compared discrete Frechet errors between the two discrete mapping methods and ground truth across all 22, 984 neuron branches in this brain sample, and found the same trend. Zeroth and first order mapping had similar deviation from ground truth using the original, complete sampling of the neurons, while there were several instances of first order mapping being superior when the neuron reconstructions were downsampled (Figure 5f–g). We downsampled the branches by retaining both end knots, along with every one out of 100 knots in between. There was a statistically significant correlation between the error difference and average sampling period in the downsampled case (Pearson’s correlation test, *α* = 0.01), but the correlation was very weak.

Further, we applied Eq. 3 and found that the error from zeroth order mapping for a line segment of length *L* microns under this transformation is bounded by, approximately 0.011*L* + 0.022. In particular, the error for a line segment of length 10 microns is at most ~ 0.13 microns and of length 1mm is at most ~ 11 microns. The observed errors in Figure 5f–g obeyed these bounds.

## Discussion

In this paper we examine the “naive” approach to mapping discretely sampled one-dimensional structures by simply transforming the positions of the knots, i.e. mapping line segments to line segments. We show that this method can be inaccurate when the Jacobian of the transformation is non-constant. We describe how to preserve derivative information which will lead to more accurate mappings in neighborhoods of the knots. We offer an implementation of a first-order mapping technique which, empirically, is more accurate on discretely sampled differentiable curves. We also apply our method to real neuron reconstructions and show that it has similar error to zeroth order mapping in the original reconstructions, but can be more accurate when the reconstructions are highly downsampled.

In our experiment with real neuron reconstructions, it is important to note what we are considering ground truth. Since the original reconstructions are in SWC format, only the knot positions are known, and the neurons are typically represented as piecewise linear structures. Real neuron morphologies are not piecewise linear, and instead are continuously curving as they pass through dense brain tissue. Nonetheless, because we have no further information about the neuron trajectories, we consider the original reconstructions to be piecewise linear, and generate the ground truth mappings by transforming the straight lines between the knots.

Since the registration transformation had a minor LDDMM component (Figure 3b), the straight lines were generally transformed into other straight lines, and the zeroth order method was therefore sufficiently accurate. We believe this result is largely driven by the different scales of the neuron reconstruction segments, and the brain registration. Neuron reconstructions were composed of knots that were spaced, typically, at the tens-of-microns scale. In contrast, brain registration was done at the scale of brain regions, which, for mice, are generally at the hundreds-of-microns scale. Indeed, the default resolution at which CloudReg performs registration is one hundred microns [6]. This means that the registration was not likely to distort reconstruction segments, making the zeroth order mapping adequate.

When neuron branches were downsampled by a factor of 100 however, larger differences in error emerged. After downsampling, the length of trace segments were long enough that the registration could warp them, as reflected by our error bounds 2, 3. If image resolution and computing power continues to improve, it is likely that registration methods will incorporate higher resolution image information. In this case, the spatial scale of registration transformations will approach the scale of neuron morphologies, rendering the original problem in Figure 2a,b more relevant, and perhaps making first order mapping useful at even smaller sampling periods.

Conversely our results can be used to make manual tracing and storage of trace data more efficient. If the registration transformation, *ϕ*, is know a priori, and there are stretches where a neuronal branch is straight, then it is possible to compute the minimum sampling rate while still controlling the amount of error introduced during mapping to atlas coordinates. For example, under the registration computed in our real data experiment, one could sample a straight branch only once every 88 microns while maintaining zeroth order mapping error below 1 micron. This is a lower sampling rate than is usually used in practice, meaning our results could make neuron tracing more efficient.

It may be tempting to use our “ground-truth” mapping method, i.e. upsampling a linear interpolation then performing zeroth order mapping, as a neuron mapping method. While this may be appropriate in some settings, this approach has two primary disadvantages. First, as stated before, neurons are not piecewise linear structures so, while the knot positions can be generally regarded as lying on the neuron, the linear interpolation cannot. Therefore, it would be necessary to keep track of which knots are from the original trace, and which knots are from the upsampling in order to preserve the original trace information. This would require existing file formats to expand their metadata conventions. Secondly, for large traces, the upsampled data could become computationally cumbersome to store.

Lastly, we want to highlight work in the adjacent field of neuron reconstruction where algorithms such as [11] can convert reconstruction knots into dense image segmentations which capture neuron trajectories at finer resolutions. Algorithms to automatically trace images of single neurons have been under development for decades [2, 14]. They could be adapted to generate both denser neuron samplings, and more accurate derivative estimates at the sampled points. These methods could improve both zeroth and first order mapping methods, so weighing these effects alongside the accuracy required for the given scientific goal would help determine which mapping method is appropriate.

## Methods

### Software Implementation

In order to implement a first order action method that transforms neuronal curves, we needed to address two questions. The first is how to estimate derivatives in the original discretely sampled curve. The second is, once the knot positions and derivatives are transformed, how can they be used to generate a continuous curve in the new space.

### One Sided Derivatives from First Order Splines

The original trace points are assumed to represent the knots in a first order spline, i.e. the points are linearly interpolated. In this representation, derivatives do not necessarily exist at the knots, but one-sided derivatives do and can be easily computed using the difference of consecutive knot positions. Both one-sided derivatives $\left(\dot{c}\left(t_{i}^{-}\right),\dot{c}\left(t_{i}^{+}\right)\right)$ are transformed according to Eq. 1 and used to generate the transformed curve.

### Fitting Curve to Transformed Positions and Derivatives

For the *i*’th curve segment, the positions *c*(*t*_*i*−1_), *c*(*t*_*i*_) and one sided derivatives $c\left(t_{i-1}^{+}\right),c\left(t_{i}^{-}\right)$ present four constraints for the interpolating curve. We use these constraints to define a cubic polynomial between each pair of knots, which is known as Hermite interpolation [10]. The result is a third order spline. Specifically, we use the scipy implementation of cubic Hermite splines [19]. It is important to note that this spline is still not necessarily differentiable at the knots.

Further details about our implementation can be found in our open-source Python package brainlit: http://brainlit.neurodata.io/.

## Supplementary Material

1

## Data Availability

The data and code used in this work is available in our open-source Python package brainlit: http://brainlit.neurodata.io/.
