# Supplementary material for: Preserving Derivative Information while Transforming Neuronal Curves
Source: ArXiv. 2023 Mar 16:arXiv:2303.09649v1. Preprint. [Version 1] (PMC10055472)
Supplement: Supplementary file 1 [file NIHPP2303.09649V1-supplement-1.pdf]

$$\phi \cdot T = \{(t_i, \phi \cdot x_i^{(k)})\}_{i=1}^{n}$$

Supplement

Mathematical Proofs

We use $|\cdot|$ to denote the Euclidean norm for elements of $\mathbb{R}^d$, and the spectral norm for matrices.

Statement 1. *For a sequence of time-stamped elements on the jet space,* $T = \{(t_i, x_i^{(k)})\}_{i=1}^{n}$ *in* $(J^k)^n$*, we define the action of diffeomorphisms*

$$\phi \cdot T = \{(t_i, \phi \cdot x_i^{(k)})\}_{i=1}^{n} \tag{1}$$

Proof. For a regular differentiable curve $c : [0, L] \to \mathbb{R}^3$ with extension $\hat{c} : [0, L] \to X^{(k)}$, we defined $\phi \cdot \hat{c}$ as the extension, $\widehat{\phi \circ c}$. In practice, only the finite sampling $\{x_i^{(k)}\}_{i=1}^n$ is accessible. However, it is always possible to define a curve c that agrees with this sampling, such as with a polynomial [12]. Then, we can apply ϕ to this curve, and compute the transformed positions and derivatives, defining the action on the finite sampling. Now, we verify the axioms of a group action.

First, the identity element (ϕ_{Id}) in the diffeomorphism group should leave a sampling unchanged. Assume that c is a curve that agrees with the sampling T:

$$\begin{aligned}
\phi_{Id} \cdot T &= \{(t_i, \bar{x}_i^{(k)}) : \bar{x}_i^{(k)} = ((\phi_{Id} \circ c)(t_i), \partial_t(\phi_{Id} \circ c)(t_i), ..., \partial_t^k(\phi_{Id} \circ c)(t_i))\}_{i=1}^n \\
&= \{(t_i, \bar{x}_i^{(k)}) : \bar{x}_i^{(k)} = (c(t_i), \partial_t c(t_i), ..., \partial_t^k c(t_i))\}_{i=1}^n \\
&= T
\end{aligned}$$

Second, a composition of diffeomorphisms ($\phi_1 \circ \phi_2$) should act successively on a sampling:

$$\begin{aligned}
(\phi_1 \circ \phi_2) \cdot T = \{&(t_i, \bar{x}_i^{(k)}) : \bar{x}_i^{(k)} = ... \\
&((\phi_1 \circ \phi_2 \circ c)(t_i), \partial_t(\phi_1 \circ \phi_2 \circ c)(t_i), ..., \partial_t^k(\phi_1 \circ \phi_2 \circ c)(t_i))\}_{i=1}^n \\
&= \{(t_i, \bar{x}_i^{(k)}) : \bar{x}_i^{(k)} = ... \\
&((\phi_1 \circ f)(t_i), \partial_t(\phi_1 \circ f)(t_i), ..., \partial_t^k(\phi_1 \circ f)(t_i))\}_{i=1}^n \qquad f \triangleq \phi_2 \circ c \\
&= \phi_1 \cdot \{(t_i, y_i^{(k)}) : y_i^{(k)} = (f(t_i), \partial_t f(t_i), ..., \partial_t^k f(t_i))\}_{i=1}^n \\
&= \phi_1 \cdot \{(t_i, y_i^{(k)}) : y_i^{(k)} = ... \\
&((\phi_2 \circ c)(t_i), \partial_t(\phi_2 \circ c)(t_i), ..., \partial_t^k(\phi_2 \circ c)(t_i))\}_{i=1}^n \\
&= \phi_1 \cdot \phi_2 \cdot \{(t_i, x_i^{(k)}) : x_i^{(k)} = (c(t_i), \partial_t c(t_i), ..., \partial_t^k c(t_i))\}_{i=1}^n \\
&= \phi_1 \cdot \phi_2 \cdot \{(t_i, x_i^{(k)}) : x_i^{(k)} = (c(t_i), \partial_t c(t_i), ..., \partial_t^k c(t_i))\}_{i=1}^n \\
&= \phi_1 \cdot \phi_2 \cdot T
\end{aligned}$$

□

Proposition 1. ***[Zeroth Order Mapping Error Bound 1]*** *Say $\phi : \mathbb{R}^3 \to \mathbb{R}^3$ is a C^1 diffeomorphism and $c : [0, L] \to \mathbb{R}^3$ is a continuous, piecewise C^1 curve parameterized by arc length with knots $\{t_i : t_1 = 0, t_n = L, t_{i-1} < t_i\}_{i=1}^n$. For the transformed curve $f = \phi \circ c$, the zeroth order mapping defines a first order spline g which satisfies:*

$$\max_{t \in [0,L]} |f(t) - g(t)| \le \delta\sqrt{3} \max_{t \in [0,L]} |D\phi \circ c(t)| \tag{2}$$

where $\delta \triangleq \max_{2 \le i \le n} |t_i - t_{i-1}|$, and $D\phi \circ c(t)$ is the Jacobian of ϕ evaluated at $c(t)$.

Proof. We will define the modulus of continuity for a function $h : [t_0, t_n] \to \mathbb{R}$ as in [10]:

$$\omega(h; \delta) = \max_{|s-t| \le \delta} |h(s) - h(t)|$$

and note that if $\dot{h}$ exists, then $|\dot{h}| \le M$ implies $\omega(h; \delta) \le M\delta$ by the mean value theorem.

The theorem on spline function approximation in [10] states that the first order spline l that interpolates the function $h : [t_0, t_n] \to \mathbb{R}$ satisfies:

$$\max_{t\in[t_0,t_n]} |l(t) - h(t)| \le \omega(h, \delta)$$

where $\delta = \max_{0\le i\le n} |t_i - t_{i-1}|$.

Now consider the curve $f = \phi \circ c$. First we will consider the curve restricted to the interval (t_i, t_{i+1}), denoted f_i. The derivative of f_i exists everywhere and is equal to $(D\phi \circ c(t)) \cdot \dot{c}(t)$, which is bounded by $\max_t |D\phi \circ c(t)|$ since $|\dot{c}(t)| = 1$ (c is parameterized by arc length).

If we split the 3D curve f_i into its coordinate functions $f(t) = (f_{i,1}(t), f_{i,2}(t), f_{i,3}(t))^T$, we note $|f_{i,k}(t)| \le |f_i(t)|$ and therefore that $\omega(f_{i,k}, \delta) \le \delta \max_t |D\phi \circ c(t)|$ for $k = 1, 2, 3$. Then for each $f_{i,k}$, we can construct with a first order spline $g_{i,k}$ from the knots $\{f_k(t_i), f_k(t_{i+1})\}$. The composite function $g_i(t) = (g_{i,1}(t), g_{i,2}(t), g_{i,3}(t))^T$ satisfies, for all $t \in (t_i, t_{i+1})$:

$$\begin{aligned}
|f_i(t) - g_i(t)| &= \sqrt{|f_{i,1}(t) - g_{i,1}(t)|^2 + |f_{i,2}(t) - g_{i,2}(t)|^2 + |f_{i,3}(t) - g_{i,3}(t)|^2} \\
&\le \sqrt{\omega^2(f_{i,1}, \delta) + \omega^2(f_{i,2}, \delta) + \omega^2(f_{i,3}, \delta)} \\
&\le \delta\sqrt{3} \max_t |D\phi \circ c(t)|
\end{aligned}$$

with $\delta = |t_{i+1} - t_i|$ since we are looking at a single segment. We can construct the full zeroth order mapping, g, by performing the same procedure over all segments indexed by i. We then maximize over all segments to get:

$$\max_{t\in[0,L]} |f(t) - g(t)| \le \delta\sqrt{3} \max_{t\in[0,L]} |D\phi \circ c(t)|$$

□

Proposition 2. ***[Zeroth Order Mapping Error Bound 2]*** *Say $\phi : \mathbb{R}^3 \to \mathbb{R}^3$ is a C^1 diffeomorphism and $c : [0, L] \to \mathbb{R}^3$ is a continuous, piecewise linear curve parameterized by arc length with knots $\{t_i : t_1 = 0, t_n = L, t_{i-1} < t_i\}_{i=1}^n$. For the transformed curve $f = \phi \circ c$, the zeroth order mapping defines a first order spline g which satisfies:*

$$\max_{t\in[0,L]} |f(t) - g(t)| \le \max_{i\in\{0,\dots,n\}, t\in[t_{i-1},t_i]} \frac{1}{2} \left(|D\phi \circ c(t) - I||t_i - t_{i-1}| + |\epsilon_i - \epsilon_{i-1}|\right) \tag{3}$$

where $\epsilon_i \triangleq c(t_i) - \phi(c(t_i))$ and $D\phi \circ c(t)$ is the Jacobian of ϕ evaluated at $c(t)$.

Proof. We will focus on a single line segment $c_i = c|_{[t_{i-1},t_i]}$, then maximize over all such segments. c_i is a function from $[t_{i-1}, t_i]$ to $\mathbb{R}^3$. Denote the endpoints of c_i as $c_{i,0} = c_i(t_{i-1})$ and $c_{i,1} = c_i(t_i)$. The zeroth order mapping of c_i defines the first order spline $g_{c_i}(t) = \phi(c_{i,0}) + \frac{(t-t_{i-1})}{t_i-t_{i-1}}(\phi(c_{i,1}) - \phi(c_{i,0}))$.

For simplicity, we will reparameterize the problem using $\sigma(t) = t_{i-1} + t(t_i - t_{i-1}) : [0, 1] \to [t_{i-1}, t_i]$ and define $c' = c_i \circ \sigma$ which is defined on $[0, 1]$. The zeroth order mapping of c' defines the spline $g_{c'}(t) = \phi(c_{i,0}) + t(\phi(c_{i,1}) - \phi(c_{i,0}))$. Note that the zeroth order mapping errors are the same in both parameterizations i.e. for $f_{c_i} = \phi \circ c_i, f_{c'} = \phi \circ c'$ we have:

$$\max_{t\in[t_{i-1},t_i]} |f_{c_i}(t) - g_{c_i}(t)| = \max_{t\in[0,1]} |f_{c'}(t) - g_{c'}(t)|$$

since, for every $t \in [0, 1]$, $|f_{c'}(t) - g_{c'}(t)| = |f_{c_i}(\sigma(t)) - g_{c_i}(\sigma(t))|$ and for every $t \in [t_{i-1}, t_i]$, $|f_{c_i}(t) - g_{c_i}(t)| = |f_{c'}(\sigma^{-1}(t)) - g_{c'}(\sigma^{-1}(t))|$. So, we have converted the problem to bounding:

$$\max_{t\in[0,1]} |f_{c'}(t) - g_{c'}(t)|$$

We have

$$f_{c'}(t) - g_{c'}(t) = \phi(c'(t)) - [\phi(c_{i,0}) + t(\phi(c_{i,1}) - \phi(c_{i,0}))]$$

and since $f_{c'}(t) - g_{c'}(t)$ vanishes at both $t = 0$ and $t = 1$, the following argument, which uses the fundamental theorem of calculus, applies both going forward from $t = 0$ and backward from $t = 1$. So, without loss of generality, we consider $0 \leq t \leq \frac{1}{2}$:

$$\begin{aligned}
f_{c'}(t) - g_{c'}(t) &= \phi(c'(t)) - [\phi(c_{i,0}) + t(\phi(c_{i,1}) - \phi(c_{i,0}))] \\
&= \int_0^t \partial_\tau \left(\phi(c'(\tau)) - [\phi(c_{i,0}) + \tau(\phi(c_{i,1}) - \phi(c_{i,0}))]\right) d\tau \\
&= \int_0^t D\phi \circ c'(\tau) \cdot \dot{c}'(\tau) - (\phi(c_{i,1}) - \phi(c_{i,0})) d\tau \\
&\leq \max_{t\in[0,1]} |D\phi \circ c'(t) \cdot \dot{c}'(t) - (\phi(c_{i,1}) - \phi(c_{i,0}))| \int_0^t d\tau \\
&\leq \frac{1}{2} \max_{t\in[0,1]} |D\phi \circ c'(t) \cdot \dot{c}'(t) - (\phi(c_{i,1}) - \phi(c_{i,0}))| \qquad\qquad t \leq \frac{1}{2} \\
&\text{Define: } \epsilon_i \triangleq c_{i,1} - \phi(c_{i,1}), \epsilon_{i-1} \triangleq c_{i,0} - \phi(c_{i,0}) \\
&= \frac{1}{2} \max_{t\in[0,1]} |D\phi \circ c'(t) \cdot (c_{i,1} - c_{i,0}) - (c_{i,1} - c_{i,0}) + (\epsilon_i - \epsilon_{i-1})| \\
&\leq \max_{t\in[0,1]} \frac{1}{2} \left(|D\phi \circ c'(t) - I||c_{i,1} - c_{i,0}| + |\epsilon_i - \epsilon_{i-1}|\right) \\
&= \max_{t\in[t_{i-1},t_i]} \frac{1}{2} \left(|D\phi \circ c(t) - I||c_{i,1} - c_{i,0}| + |\epsilon_i - \epsilon_{i-1}|\right) \\
&= \max_{t\in[t_{i-1},t_i]} \frac{1}{2} \left(|D\phi \circ c(t) - I||t_i - t_{i-1}| + |\epsilon_i - \epsilon_{i-1}|\right)
\end{aligned}$$

where the last equality comes from the fact that c is parametrized by arc length, so $|t_i - t_{i-1}| = |c_{i,1} - c_{i,0}|$. In summary we have:

$$\max_{t\in[t_{i-1},t_i]} |f_{c_i}(t) - g_{c_i}(t)| \leq \max_{t\in[t_{i-1},t_i]} \frac{1}{2} \left(|D\phi \circ c(t) - I||t_i - t_{i-1}| + |\epsilon_i - \epsilon_{i-1}|\right)$$

Finally, we maximize over all segments to get:

$$\max_{t\in[0,L]} |f(t) - g(t)| \leq \max_{i\in\{0,\ldots,n\},t\in[t_{i-1},t_i]} \frac{1}{2} \left(|D\phi \circ c(t) - I||t_i - t_{i-1}| + |\epsilon_i - \epsilon_{i-1}|\right)$$

where $f = \phi \circ c$ and g is the first order spline defined by the zeroth order mapping of c.

□

Proposition 3. ***[Comparable Bounds for Zeroth and First Order Mapping]*** *Say $\phi : \mathbb{R}^3 \rightarrow \mathbb{R}^3$ is a C^4 diffeomorphism and $c : [a, b] \rightarrow \mathbb{R}^3$ is a continuous, piecewise C^4 curve parameterized with knots $\{t_i : t_1 = a, t_n = b, t_{i-1} < t_i\}_{i=1}^n$. For the transformed curve $f = \phi \circ c$ defined by coordinate functions $f = (f^0, f^1, f^2)^T$, the zeroth order mapping defines a first order spline g_0 which satisfies:*

$$\max_{t\in[a,b]} |f(t) - g_0(t)| \leq \frac{\sqrt{3}}{4} \max_{t\in[a,b],j\in\{0,1,2\}} |\partial_t^{(4)} f^j(t)| \left(\frac{\delta}{2}\right)^4 + \frac{\sqrt{3}}{2}\left(\frac{\delta}{2}\right)^2 \max_{i\in\{1\dots n\},j\in\{0,1,2\}} |\partial_t^{(3)} f^j(t_i)| \left(\frac{\delta}{2}\right) + \frac{\sqrt{3}}{2}\left(\frac{\delta}{2}\right)^2 \max_{i\in\{1\dots n\},j\in\{0,1,2\}} |\partial_t^{(2)} f^j(t_i)| \quad (4)$$

where $\delta \triangleq \max_{2\leq i\leq n} |t_i - t_{i-1}|$ and $\partial_t^{(k)} f^j(t)$ is the k'th derivative of f^j evaluated at t. Also, the first order mapping defines a third order spline g_1, which satisfies

$$\max_{t\in[a,b]} |f(t) - g_1(t)| \leq \frac{\sqrt{3}}{4!} \max_{t\in[a,b],j\in\{0,1,2\}} |\partial_t^{(4)} f^j(t)| \left(\frac{\delta}{2}\right)^4 \quad (5)$$

and we note that the bound in 5 is tighter than the bound in 4. Further, there exists a transformed curve f and a set of knots $\{t_i\}_{i=1}^n$ that achieves both bounds exactly.

Proof. We will prove both bounds starting with a single segment of c, then extending to the entire piecewise curve.

The bound in 5 comes from the error estimate of Hermite interpolation for one dimensional functions, Theorem 2 in Section 6.3 of [10]. For a single segment between knots t_{i-1}, t_i, this theorem states that if p is the polynomial of degree at most 3 which agrees with h and $\partial_t h$ at the knots, then, for each t, there exists a point $\xi \in (t_{i-1}, t_i)$ such that:

$$h(t) - p(t) = \frac{\partial_t^{(4)} h(\xi)}{4!}(t - t_{i-1})^2(t - t_i)^2$$

therefore, for all t

$$|h(t) - p(t)| \leq \frac{1}{4!} \max_{t\in[t_{i-1},t_i]} |\partial_t^{(4)} h(t)| \left(\frac{t_i - t_{i-1}}{2}\right)^4$$

The first order mapping is indeed the third order spline that matches function and derivative values at the knots, so the above bound applies to all three coordinate functions of $f = (f^0, f^1, f^2)^T$ and $g_1 = (g_1^0, g_1^1, g_1^2)^T$. To accommodate all three dimensions, we maximize over all dimensions and add a $\sqrt{3}$ term:

$$\max_{t\in[t_{i-1},t_i]} |f(t) - g_1(t)| \leq \frac{\sqrt{3}}{4!} \max_{t\in[t_{i-1},t_i],j\in\{0,1,2\}} |\partial_t^{(4)} f^j(t)| \left(\frac{t_i - t_{i-1}}{2}\right)^4$$

Then if we maximize both sides over all the segments, we get

$$\max_{t\in[a,b]} |f(t) - g_1(t)| \leq \frac{\sqrt{3}}{4!} \max_{t\in[a,b],j\in\{0,1,2\}} |\partial_t^{(4)} f^j(t)| \left(\frac{\delta}{2}\right)^4$$

The bound in 4 comes from the error estimate of polynomial interpolation, Theorem 2 in Section 6.1 of [10]. This theorem states that, for a single segment between knots t_{i-1}, t_i, if p is the line that agrees with a one dimensional function h at the knots, then there exists a point $\xi \in (t_{i-1}, t_i)$ such that:

$$h(t) - p(t) = \frac{\partial_t^{(2)} h(\xi)}{2}(t - t_{i-1})(t - t_i)$$

therefore

$$\max_{t\in[t_{i-1},t_i]} |h(t) - p(t)| \leq \frac{1}{2} \max_{t\in[t_{i-1},t_i]} |\partial_t^{(2)} h(t)| \left(\frac{t_i - t_{i-1}}{2}\right)^2 \tag{6}$$

Our remaining task is to relate the maximum second derivative to the maximum fourth derivative. We start by using the fundamental theorem of calculus twice to get (for $t \in [t_{i-1}, t_i]$):

$$\partial_t^{(2)} h(t) = \int_{t_{i-1}}^{t} \int_{t_{i-1}}^{\tau} h^{(4)}(v) dv d\tau + \partial_t^{(3)} h(t_{i-1})(t - t_{i-1}) + \partial_t^{(2)} h(t_{i-1})$$

therefore

$$\begin{aligned} |\partial_t^{(2)} h(t)| \leq & \max_{t\in[t_{i-1},t_i]} |\partial_t^{(4)} h(t)| \int_{t_{i-1}}^{t} \int_{t_{i-1}}^{\tau} dv d\tau + \\ & |t - t_{i-1}| \max_{t\in\{t_{i-1},t_i\}} |\partial_t^{(3)} h(t)| + \max_{t\in\{t_{i-1},t_i\}} |\partial_t^{(2)} h(t)| \\ & = \frac{1}{2}(t - t_{i-1})^2 \max_{t\in[t_{i-1},t_i]} |\partial_t^{(4)} h(t)| + \\ & |t - t_{i-1}| \max_{t\in\{t_{i-1},t_i\}} |\partial_t^{(3)} h(t)| + \max_{t\in\{t_{i-1},t_i\}} |\partial_t^{(2)} h(t)| \end{aligned} \tag{7}$$

Similarly,

$$\partial_t^{(2)} h(t) = \int_{t_i}^{t} \int_{t_i}^{\tau} h^{(4)}(v) dv d\tau + \partial_t^{(3)} h(t_i)(t - t_i) + \partial_t^{(2)} h(t_i)$$

therefore

$$\begin{aligned} |\partial_t^{(2)} h(t)| \leq & \max_{t\in[t_{i-1},t_i]} |\partial_t^{(4)} h(t)| \int_{t_i}^{t} \int_{t_i}^{\tau} dv d\tau + \\ & |t - t_i| \max_{t\in\{t_{i-1},t_i\}} |\partial_t^{(3)} h(t)| + \max_{t\in\{t_{i-1},t_i\}} |\partial_t^{(2)} h(t)| \\ & = \frac{1}{2}(t - t_i)^2 \max_{t\in[t_{i-1},t_i]} |\partial_t^{(4)} h(t)| + \\ & |t - t_i| \max_{t\in\{t_{i-1},t_i\}} |\partial_t^{(3)} h(t)| + \max_{t\in\{t_{i-1},t_i\}} |\partial_t^{(2)} h(t)| \end{aligned} \tag{8}$$

So, we have two bounds for $|\partial_t^{(2)} h(t)|$, given by 7 and 8. Both bounds are in the form of a second order polynomial of t, so it is straightforward to show that the combined bound is maximal at the midpoint between t_{i-1} and t_i, where the bounds also happen to intersect. Thus, we have:

$$\begin{aligned} \max_{t\in[t_{i-1},t_i]} |\partial_t^{(2)} h(t)| \leq & \frac{1}{2}\left(\frac{t_i - t_{i-1}}{2}\right)^2 \max_{t\in[t_{i-1},t_i]} |\partial_t^{(4)} h(t)| + \\ & \left(\frac{t_i - t_{i-1}}{2}\right) \max_{t\in\{t_{i-1},t_i\}} |\partial_t^{(3)} h(t)| + \max_{t\in\{t_{i-1},t_i\}} |\partial_t^{(2)} h(t)| \end{aligned} \tag{9}$$

Combining 9 with 6, we get

$$\max_{t\in[t_{i-1},t_i]}|h(t)-p(t)| \leq \frac{1}{4}\max_{t\in[t_{i-1},t_i]}|\partial_t^{(4)}h(t)|\left(\frac{t_i-t_{i-1}}{2}\right)^4 +$$
$$\frac{1}{2}\left(\frac{t_i-t_{i-1}}{2}\right)^3\max_{t\in\{t_{i-1},t_i\}}|\partial_t^{(3)}h(t)|+$$
$$\frac{1}{2}\left(\frac{t_i-t_{i-1}}{2}\right)^2\max_{t\in\{t_{i-1},t_i\}}|\partial_t^{(2)}h(t)|$$

Again, to extend this result from a function that takes values in $\mathbb{R}$, to one that takes values in $\mathbb{R}^3$, we need to maximize over all dimensions and include a factor of $\sqrt{3}$. After maximizing over all segments, we get, for a piecewise C^4 curve $f = \phi \circ c : [a,b] \to \mathbb{R}^3$, and the zeroth order mapping g_0 which linearly interpolates the mapped knots:

$$\max_{t\in[a,b]}|f(t)-g_0(t)| \leq \frac{\sqrt{3}}{4}\max_{t\in[a,b],j\in\{0,1,2\}}|\partial_t^{(4)}f^j(t)|\left(\frac{\delta}{2}\right)^4 +$$
$$\frac{\sqrt{3}}{2}\left(\frac{\delta}{2}\right)^2\max_{i\in\{0\ldots n\},j\in\{0,1,2\}}|\partial_t^{(3)}f^j(t_i)|\left(\frac{\delta}{2}\right)+$$
$$\frac{\sqrt{3}}{2}\left(\frac{\delta}{2}\right)^2\max_{i\in\{0\ldots n\},j\in\{0,1,2\}}|\partial_t^{(2)}f^j(t_i)|$$

Finally, we note that if the mapped curve is $f(t) = (1-t^2, 1-t^2, 1-t^2)^T$ defined on $[-1,1]$ with two knots $t_0 = -1, t_1 = 1$, then the zeroth order mapping is $g_0(t) = (0,0,0)$ and the first order mapping is $g_1(t) = (1-t^2, 1-t^2, 1-t^2)$. The first order mapping bound satisfies the bound from 4 of 0, because $f = g_1$. The zeroth order bound is $\sqrt{3}$ which is achieved by the zeroth order mapping at $t = 0$. □
